# Supplementary material for: “I’ve not been knocked out, so I’ll probably be fine:” Amateur Rugby players do not know the risks they are taking
Source: PLoS One. 2025 Jun 4;20(6):e0325368. doi: 10.1371/journal.pone.0325368 (PMC12136326; doi:10.1371/journal.pone.0325368)
Supplement: Attachment — Submitted filename: Responses_and_Amendments (1).docx [file pone.0325368.s002.docx]

Manuscript ID PONE-D-24-36156 titled ““I’ve not been knocked out, so I’ll probably be fine.” Amateur Rugby players do not know the risk they are taking: This needs to change.”

**PLOS ONE**

The authors have attempted to address the reviewer’s suggestions as highlighted below.

| **Reviewer comments to Authors** |  |
| --- | --- |
| **Comment/Recommendation** | **Amendment** |
| Thank you for the opportunity to review this paper. Whilst I find the paper interesting and the results potentially insightful and potentially useful for driving future work in the area, I feel there are some major problems with the manuscript in its current format. Firstly, some key pieces of up-to-date literature are missing from the introduction. Secondly no discussion of where the findings is provided. The current discussion sections reads as if it were the results and there are no further comments of where the authors findings sit within the current body of literature. Inclusion of some more recent references is required and use of the information within these should be used to form a discussion to create a more detailed view of where this research sits in the field and the impact it has. | Thank you for taking the time to review our manuscript, and for the positive comments you have provided.  We have included a detailed discussion that situates our findings and study within the current literature. We refer to where our findings compliment the literature, and further it. |
| Introduction L52; Please also consider including the most up to date rugby union concussion incidence data reported in Williams et al (doi; 0.1007/s40279-021-01603-w) | Thank you for this comment. We have amended this to include updated incidence rates. |
| L87; Please consider the use of and information from Tadmore et al (doi; 10.1016/j.jsams.2023.09.015), Tadmore et al. (doi; 10.1136/ip-2023-045108) and Longworth et al (doi; 10.1136/bmjsem-2020-000860) to further detail and discuss why players may under report concussions. | Thank you for this comment. We have read through the work of Tadmor et al. (2023), Tadmor et al. (2024), and Longworth et al. (2021) and have incorporated the findings from these studies into our work. We believe this has added great value to our work. |
| Practical Implications L239; Walker at al (2023) does not appear on the reference list. This is also a very bold statement and would be wary of including this in the paper without serious critique of the findings from Walker et al. Would it be more impactful to discuss more structured concussion education that already goes on in the RFU or world rugby and how it is disseminated, rather than just focussing on a point around depression. | Thank you for this comment. We have decided to remove the “Practical Implications” section as your advice to refer to RFU and World Rugby concussion guidelines have been discussed in the Discussion section. |
