# Supplementary material for: “I’ve not been knocked out, so I’ll probably be fine:” Amateur Rugby players do not know the risks they are taking
Source: PLoS One. 2025 Jun 4;20(6):e0325368. doi: 10.1371/journal.pone.0325368 (PMC12136326; doi:10.1371/journal.pone.0325368)
Supplement: Attachment — Submitted filename: Review PLOSONE.docx [file pone.0325368.s001.docx]

**Dear Editors and Authors,**

Thank you for the opportunity to review this manuscript. Below is my detailed review, including comments on methodology, discussion, and ethical considerations. I hope my feedback is helpful in improving the paper.

## **Overall Assessment**

This manuscript addresses an important and timely issue in sports medicine and concussion awareness in amateur rugby. The use of qualitative interviews is an appropriate method to explore the attitudes and knowledge of players regarding concussion risks. The manuscript is generally well-structured and presents clear findings. However, there are several areas where improvements can be made, particularly in **contextualizing the results within the existing literature, refining the discussion, and addressing ethical concerns regarding data accessibility**.

## **Detailed Review**

### **1. Title and Abstract**

**Strengths:**

- The title is engaging and effectively captures the study’s focus.
- The abstract concisely summarizes the study’s aims, methods, and key findings.

**Areas for Improvement:**

- The study’s objective should be more explicitly stated in the abstract. It is currently implied that the study investigates a lack of knowledge, but this could be clearer.
- Consider including a more direct mention of the study’s practical implications (e.g., policy, education, or intervention strategies).

### **2. Introduction**

**Strengths:**

- The introduction effectively situates the study within the broader context of concussion risks in rugby.
- The motivation for the study is clear.

**Areas for Improvement:**

- Although the authors have incorporated additional recent studies after previous review comments, **the knowledge gaps that this study aims to address should be more explicitly stated**.
- The discussion of concussion incidence in rugby compared to other sports (football, American football) could be further elaborated.
- While recent studies (e.g., Tadmor et al., 2023; Longworth et al., 2021) have been integrated, it would be helpful to explicitly state what gaps remain in the literature.

### **3. Methodology**

**Strengths:**

- The use of **reflexive thematic analysis** is appropriate for the research question.
- The study provides transparency by outlining the authors’ positioning in the research.

**Areas for Improvement:**

- **The sample size (n=9) is small** and limited to England. While qualitative research does not require large samples, **a brief discussion of how this affects generalizability** could be included in the methods section.
- **The rationale for selecting participants with at least three concussions** should be explained further. Could including players with fewer concussions provide additional insight?
- The authors discuss their **own positionality**, which is commendable, but **potential biases in data collection and interpretation should also be acknowledged**.

### **4. Results**

**Strengths:**

- The two main themes (**"Poor Duty of Care"** and **"Lack of Education"**) are well-developed and supported by participant quotes.
- The authors provide a **strong narrative** illustrating players’ lived experiences.

**Areas for Improvement:**

- Consider **exploring subthemes** within the two overarching themes to provide more nuance.
- Were there any **differences in responses between male and female participants**? If so, these should be discussed.
- A **visual representation** (e.g., thematic map or summary table) could help clarify the findings.

### **5. Discussion**

**Strengths:**

- The study is well-integrated with existing literature.
- The discussion of **policy implications and the role of governing bodies** is relevant.

**Areas for Improvement:**

- Some sections of the discussion **read more like results rather than analysis**. A deeper interpretation of findings in relation to past studies is needed.
- The **study’s unique contribution** compared to previous qualitative research should be **more explicitly stated**.
- The discussion about **RFU and World Rugby concussion education programs** is useful, but **specific suggestions for improvement** should be provided.

### **6. Ethical Concerns Regarding Data Accessibility**

One **major concern** is that **all interview transcripts are openly available online**. While transparency is valuable, **this raises significant ethical and data protection issues**:

- **Confidentiality risks**: Even if anonymized, participants' experiences might be recognizable to teammates, coaches, or clubs, leading to potential social consequences.
- **GDPR and ethical compliance**: Under data protection regulations (e.g., GDPR), even **indirectly identifiable information** (e.g., age, gender, club affiliations) could pose privacy risks.
- **Risk of data misuse**: Open-access qualitative data can be **taken out of context** or used in ways that the researchers did not anticipate.

### **Recommendation:**

- **Restrict access to transcripts** through a **controlled repository**, requiring researchers to apply for access.
- **Ensure full anonymization** before data sharing.
- **Clarify in the manuscript** why the transcripts were made publicly available and how participant confidentiality was ensured.

This ethical concern should be addressed **before publication**.

### **7. Limitations and Future Research**

**Strengths:**

- The study **acknowledges key limitations**, including sample size and demographic constraints.
- The suggestion for **future research to include a broader sample** is appropriate.

**Areas for Improvement:**

- Consider recommending **longitudinal studies** to track whether educational interventions lead to lasting changes in concussion attitudes.
- A brief discussion of **cultural differences** in concussion management between different rugby nations (UK, South Africa, New Zealand) could strengthen the study’s conclusions.

### **8. References**

**Strengths:**

- Most key studies are cited.
- Additional references have been incorporated after the previous review.

**Areas for Improvement:**

- Ensure **all cited studies are listed in the reference section** (e.g., Walker et al., 2023 was missing previously).
- Incorporate additional **recent** studies (2023–2024) where relevant.

## **Final Recommendation: Minor Revisions Required**

This study makes an **important contribution** to concussion research in amateur rugby. However, **minor revisions** are necessary before publication. The most critical issue is the **open accessibility of interview transcripts**, which **must be addressed** to comply with ethical and data protection standards. Additionally, **clarifications in the methodology, discussion, and references** would strengthen the paper.

### **Summary of Key Revisions Needed**

✅ **Clarify the study’s objective in the abstract.**

✅ **Better define knowledge gaps in the introduction.**

✅ **Discuss sample selection and generalizability in the methodology.**

✅ **Provide deeper analysis in the discussion (e.g., gender differences, policy implications).**

✅ **Consider adding a thematic map or summary table.**

✅ **Address serious ethical concerns regarding open access to interview transcripts.**

✅ **Ensure all references are correctly listed and updated.**

Once these issues are addressed, the manuscript will be in a strong position for publication.
