# Supplementary material for: “I’ve not been knocked out, so I’ll probably be fine:” Amateur Rugby players do not know the risks they are taking
Source: PLoS One. 2025 Jun 4;20(6):e0325368. doi: 10.1371/journal.pone.0325368 (PMC12136326; doi:10.1371/journal.pone.0325368)
Supplement: Attachment — Submitted filename: Responses_and_Amendments.docx [file pone.0325368.s003.docx]

Manuscript ID PONE-D-24-58092 titled ““I’ve not been knocked out, so I’ll probably be fine.” Amateur Rugby players do not know the risk they are taking: This needs to change.”

**PLOS ONE**

The authors have attempted to address the reviewer’s suggestions as highlighted below.

| **Reviewer 1 comments to Authors** | | |
| --- | --- | --- |
| **Comment/Recommendation** | **Amendment** | **Location** |
| This study makes an important contribution to concussion research in amateur rugby. However, minor revisions are necessary before publication. The most critical issue is the open accessibility of interview transcripts, which must be addressed to comply with ethical and data protection standards. Additionally, clarifications in the methodology, discussion, and references would strengthen the paper | Thank you for taking the time to review our article. We have addressed all comments made and made amendments and provided responses below. | - |
| Clarify the study’s objective in the abstract | Thanks for this comment, we have added a sentence to the abstract that clarifies this further. | L21-35. |
| Better define knowledge gaps in the introduction | Thank you for this comment. We have re-worked relevant areas of the introduction. | L44-142 |
| Discuss sample selection and generalizability in the methodology | Thank you for this comment. We have re-worked relevant areas of the method. | L143-209 |
| Provide deeper analysis in the discussion (e.g., gender differences, policy implications) | Thank you for this comment. We have now re-worked the discussion section to account for your suggestions. | L307-450 |
| Consider adding a thematic map or summary table | Thanks for this comment. As our quotes are lengthy, we feel that a thematic map would not serve the reader. We purposefully indented the quotes to differentiate from the surrounding narrative for ease of navigation in contrast to analyses whereby quotes are embedded within. | - |
| Address serious ethical concerns regarding open access to interview transcripts | Thank you for this comment. As researchers dedicated to open access research, we decided that all interview data would be made available in the interests of transparency. This is a standard practice at our university, and at the journal we are submitting to, and serves the reader should they wish to understand our conclusions. | - |
| Ensure all references are correctly listed and updated | Thank you for this comment. These have now been amended. | L509-736 |
| This manuscript addresses an important and timely issue in sports medicine and concussion awareness in amateur rugby. The use of qualitative interviews is an appropriate method to explore the attitudes and knowledge of players regarding concussion risks. The manuscript is generally well-structured and presents clear findings. However, there are several areas where improvements can be made, particularly in contextualizing the results within the existing literature, refining the discussion, and addressing ethical concerns regarding data accessibility | Thank you for this comment and for taking the time to read our manuscript. We feel as though the comments you have given have added significant value to our paper. | - |
| **Reviewer 2 comments to Authors** | | |
| **Comment/Recommendation** | **Amendment** | **Location** |
| This paper discusses a pertinent issue in sport, however, there is a need to further discuss the findings and methods in more detail. Some content within this paper is written in sensationalist/colloquial language, which should be toned down for publication in the scientific context. A focus on “referenceable ” sources should be maintained and more detail is needed within the methods and results. Please find some comments below that I hope will support the development of this work. | Thank you for taking the time to review our article. We have addressed all comments made and made amendments and provided responses below. | - |
| Introduction Line 60 -62 arguably in the Miller example there is no reference to the reasons behind the non-reporting. I think this sentence needs a link between them , and into the Fraas reference on line 66. Again on line 67 any assessment of knowledge/ its relationship with behaviour in the Miller ref is not discussed. | Thanks for this comment. Miller et al. (2021) state that the reasons for not reporting concussion are not considering it a serious injury, not knowing that it was concussion at the time, and wanting to continue participating. These were found in American Rugby players. The same was found in Irish Rugby players by Fraas et al. (2014). Therefore, the point we are making here is that these key reasons are consistent across rugby across continents and are therefore probably likely in English rugby players too. I see your point that neither study explicitly refers to attitudes and knowledge, however, these are our observations. If players are not reporting concussion due to not considering it a serious injury, not knowing that it was concussion at the time, and wanting to continue participating then we believe it is due to poor attitudes poor knowledge or both. | - |
| From line 86 concussion education/ knowledge is discussed, however I feel this section would benefit from discussion of more recent literature – the world rugby team (Danielle salmon etc) have published some relevant work on this. | Good point! We have now included Salmon et al. (2021) in this paragraph and feel it further substantiates the point being made. Thank you. | L 96-100 |
| In the introduction, there is little discussion of the existing literature that reports lived experience of concussion education and how it may influence experience, despite this being the focus of the paper. I feel this section would be strengthened by greater discussion of this, and the point that survey’s only capture a snapshot should be made stronger. In line 106, I feel the aim should be flipped so that the value of qualitative investigation to capture the rich/complexity and transiency of concussion symptoms/ lived experience, is highlighted, rather than a more vague reference to the RoCKAS as inconsistent. This paper adds value to the often numbers based literature on this topic, and its important to highlight that! | Thank you for this comment. We have now added a paragraph that follows on from the issues with ROCKAS that argues for the strengths of a qualitative approach in this domain. We have also amended the concluding paragraph in the introduction to reflect this and improve readability. | L110-119 |
| Method Line 117 please explain this point further, at present I am not sure what this means – highly individual perhaps? | Thank you for this comment. The researchers adopted this ontological view as the interpretivist paradigm allows subjective experiences to be explored within their social context. We have amended the manuscript to reflect this. | L149-153 |
| In the situating the authors paragraph 122, positionality could be discussed in greater depth. The authors are described, but how did the author position influence interview data? The phrase “the authors are motivated for others to adopt a similar outlook” almost implies the authors have an agenda, or may ask leading questions. I’d suggest rewording this more neutrally, or detailing explicitly how this motivation may specifically influence the interviews. Do the authors have rugby playing experience? | Thanks for this comment. We would agree here that positionality is important in the context of qualitative research but argue that it is unavoidable. There is certainly the ambition from the researchers to inform rugby players of the true dangers of concussion and to support their decision making process on a global scale of the sport. However, having made the interview transcripts openly available, yourself and the reader are able to view and assess the questions asked by the researcher, of which we believe none are leading. | - |
| If this data were collected, it would be more relevant to report ps gender, rather than sex, given the gendered experiences of concussion referenced within existing literature and discussion of social, rather than biological phenomena. If you don’t have this data, a sentence added to explain this would be beneficial. Please discuss how/why a sample of 9 participants was used – was there any screening, how many people expressed interest in taking part? | Only nine participants expressed interest in being interviewed for this study and were therefore included. Gender data was not obtained, rather biological sex was obtained due to this distinction in participating (male/female rugby). | - |
| Please could you discuss the topics discussed during the interview in more detail and make the interview guide publicly available. Are all the interview topics relevant to concussion reported in the results? | Thanks for this comment. We have now added the interview guide to OSF alongside interview transcripts to aid the readers’ interpretation of our findings. | - |
| If ethics would allow, and information were available from transcripts, greater participant information would be valuable in table 1, such as playing level (amateur is still quite broad, club/ university/ urban/ rural/ men’s/ women’s cultures can be different ), playing experience etc. A graphic explaining each ps’ characteristics would be a nice visual of who the sample represents | Thanks for this valuable comment. We have now added a Participant characteristics Table 2 that presents more information of each participant, their age at the time of interview, how long has passed since their last concussion, how long they have played rugby for and at what competitive levels. | - |
| Reference to timelines should be added – are the Ps current players, how long ago were the concussions? This is important given that attitudes in sport change with time, as much detail re timings of playing/ concussion should be given. | Thanks for this comment. We agree and hope that the information we have added in Table 2 satisfies this request and improves the readers’ interpretation of our data. | - |
| The limitations section mentions that one of the sample has had 20 concussions – this is very important, but isn’t referenced in the findings or participant characteristics. | Thanks for this comment. We had previously included this data in Table 1. where we highlight the range of the number of concussions sustained as well as the M and SD for this. | - |
| Data analysis What measures were undertaken to maintain the rigor/ quality of the analysis? Braun and Clarke have got a quality checklists (published 2019 off the top of my head) that could be applied and referenced here. | Thanks for this comment. We have now included reference to Braun & Clarke’s 15-point checklist. | L202-206 |
| Findings I think the results section could be expanded to further described the nuance of each situation and whether experiences were shared or diverse amongst ps with different characteristics. Greater presentation of participant quotes in-text/ in a supporting table would be beneficial- Are all ps represented meaningfully in the findings? | Thanks for this comment. Themes were developed based on shared experiences, and the quotes provided for each participant are to demonstrate these shared experiences that substantiate each theme. All participants feature in the article and therefore are represented fairly. | - |
| Are all relevant topics listed in the interview description presented in the findings? – Did concussion history/competitiveness affect attitudes? Were participant perspectives different, or consistent amongst each other. | Thanks for this comment. We appreciate the outlook of concussion history and competitiveness affecting attitudes; however, we would argue this seems to be a more quantitative approach. For example, we can estimate that concussion attitudes may be worse following a concussion (i.e. continuing participation with a concussion / continuing playing rugby generally once concussion symptoms have subsided) however, we cannot be sure based on the current methodology. This seems to be more aligned with a quantitative approach (e.g., assessing attitudes before and after a concussion occurs).  Regarding the consistency of participant perspectives, each theme was substantiated by consistency across the dataset. | - |
| A thematic map with example quotes, or a pen profile (e.g <https://link.springer.com/article/10.1186/1471-2458-11-831>) would benefit the results section. Could any links be made between knowledge and attitudes – does one preceed/influence the other? | Thanks for this comment. As our quotes are lengthy, we feel that a thematic map would not serve the reader. We purposefully indented the quotes to differentiate from the surrounding narrative for ease of navigation in contrast to analyses whereby quotes are embedded within. | - |
| Discussion I think the discussion could be strengthened with greater references to broader literature, and discussion of the study’s results/ players experiences, rather than references essentially to the theme titles. | Thank you for this comment. We feel this has been properly addressed with the reworking of the discussion section. | L307-451 |
| “For one to state that they are aware of potential neurodegenerative diseases and to choose to continue participating in Rugby, would lead one to believe they do not fully understand the risk, and in-fact have poor knowledge or negative concussion attitude.”- This reads in quite a sensationalist manner - arguably there is a big jump between concussion awareness/ appropriate management and awareness of the link between neurodegenerative disease here. This point could be supported by work that highlights that concussion knowledge doesn’t always lead to attitude change, and more recent literature generally could be cited in this chapter. Does the Tadmor paper on 278 state why players would dissuade family members or describe the concussion knowledge of the sample and link it to this point? In the current writing I’m not convinced that the Tadmor reference is able to support the previous sentence. | Thank you for this comment. We agree and have now amended this paragraph. The initial sentence was perhaps a little provocative and we have changed this along with the nature of the succeeding argument. Instead, now we discuss how health-related behaviour change is tricky and how this relates to concussion in Rugby, whereby Tadmor et al (2023) is an appropriate reference. | L308-328 |
| This discussion could present a more critical references to the literature, there is a fair amount of research that discusses responsibility in concussion and the bystander effect which would add value here. Gender differences have been cited in concussion experience/ management, are your findings consistent with that literature?`. Some sweeping statements are made without link to references, or acknowledgement as grey/anecdotal sources. | Thank you for this comment. We have thoroughly re-worked the discussion section with the addition of relevant literature and concussion guidelines practices. | L307-451 |
| Line 283 – this paragraph could be expanded with more detail. Is the bystander effect relevant here, there are some papers (by Danielle salmon I think) that reference this in rugby. | Thank you for this comment. We have added more detail here regarding a bystander effect and what this means for Rugby and the current study. | L329-337 |
| 295 colloquial language here, try to avoid. Are there any references/ theories that could back up this next sentence? The authors interpret that poor duty of care is due to lack of education, but did your sample offer their perspectives on this? | We asked questions regarding education as the interview guide states which we have now added to OSF. This was asked in a way that gave participants the opportunity to tell the researcher the level of education they had received from their club. We felt we could not ask directly whether lack of education led to poor duty of care as we believe this would have been leading. | - |
| 317 this reads as an opinion, I think there would be more value describing the current landscape of attitudes, rather than what should happen in an ideal world. Or instead, add a reference to some coach/ referee policy/code of conduct that references responsibility/duty of care. | Thanks for this comment. We have now added the coaches’ code of conduct and role of match officials set out by World Rugby that highlights their responsibilities to keep players safe, and how our study demonstrates the opposite. | L379-400 |
| Line 327 this paragraph could be supported by wider references. The Chris nowinski bradford hill paper would be relevant here as would a further explanation of the point made in lines 329-331. Can you quantify/explain further how the Walker 23 paper “partially rectifies this” ? | Thanks for this comment. This is a good point and we have added this into this paragraph. We feel this has added value to the point we were making here. | L401-412 |
| In 336 I am struggling to understand what comparison is being made. 341-43 is a sweeping statement, could existing literature that discusses access to concussion information be discussed instead – just because the documents are passively available doesn’t mean players make efforts to access them! | Thanks for these comments. We have rephrased the beginning of this paragraph now to make it explicit that the comparison we are making is that Walker et al (2023) attempt to provide a simple easily digestible message to players whereas the World Rugby guidance is a comprehensive 10-page document, that sceptics may suggest is unlikely to be read or engaged with by players. | L401-402 |
| 343 – colloquial/sensationalist language used. Could these media articles be referenced? Broader reference to the representation of concussion in the media could be made, or the the capacity of the media to influence behaviour? | Thanks for these comments. We have now removed this sentence from the manuscript during our re-working of the discussion. | - |
| 357 – I am not sure of the concept of full education, could this be worded differently. 358 Can this be backed up with theory/references to strengthen it? Is the general lack of pitch-side healthcare in amateur sport relevant here / in the discussion generally- ? | Thanks for this comment. We have changed the word “fully” to “appropriately” and deem this the correct phrasing to illustrate our point.  On the second point, we have now incorporated some discussion on the Health Belief Model and how this relates to Rugby and concussion. Evidence that HBM is linked with positive concussion attitudes is also discussed. | L429-451 |
| Limitations A small rich sample of data is a strength here rather than a limitation, and adds missing richness to often quantitative investigations of this topic. Would suggest framing as such! | Thanks for this comment. We feel this paragraph is appropriate in acknowledging that our sample is lower than other qualitative studies that have investigated similar. We have defended the decision to use a qualitative approach and the benefits that it possesses over quantitative methods. | - |
| Its surprising that more ps details are offered for the first time in the limitations section – particularly for the ps with over 20, this information would be very useful referenced in the ps characteristics/ results. | Thanks for this comment. We had previously included this data in Table 1. where we highlight the range of the number of concussions sustained as well as the M and SD for this. | - |
| 382 begins as a limitation, but as the paragraph develops, I feel it could be incorporated in the discussion instead. | Thanks for this comment. We agree this makes sense to add into the discussion and we have done so. We have retained this as a limitation but complimenting what has been referred to in the discussion. | - |
| Conclusion 401- I think this should be reframed, what would be a “full” education? Awareness of current guidelines instead. This conclusion seems too broad given the sample included 9 players, yet it speaks for UK amateur players as a whole. | Thanks for this comment. We have restructured the conclusion now to reflect the advice given. | L483-496 |
| This paper has great potential. I hope the comments provided will strengthen this work | Thank you for this positive comment. We feel that your comments and observations have improved this manuscript significantly. | - |
