# Supplementary material for: “I’ve not been knocked out, so I’ll probably be fine:” Amateur Rugby players do not know the risks they are taking
Source: PLoS One. 2025 Jun 4;20(6):e0325368. doi: 10.1371/journal.pone.0325368 (PMC12136326; doi:10.1371/journal.pone.0325368)
Supplement: Attachment — Submitted filename: Responses_and_Amendments_auresp_2.docx [file pone.0325368.s004.docx]

Manuscript ID PONE-D-24-58092 titled ““I’ve not been knocked out, so I’ll probably be fine.” Amateur Rugby players do not know the risks they are taking”

**PLOS ONE**

The authors have attempted to address the reviewer’s suggestions as highlighted below.

| **Reviewer 3 comments to Authors** | | |
| --- | --- | --- |
| **Comment/Recommendation** | **Amendment** | **Location** |
| The authors conducted qualitative research of awareness and attitude towards concussion on 9 amateur rugby players. Through reflexive thematic analysis, “Poor duty of care” and “lack of education” were found to be the main reason for encouraging continued participation from the player with suspected concussion. The findings were potentially important considering the high prevalence of concussion in British amateur Rugby. | Thank you for this comment. We appreciate the kind feedback of our work. | - |
| One worry about the findings is that the conclusions could be biased from several aspects. The subjects could volunteer to participate in the study for certain biased reasons. Even though qualitative research is less influenced by numbers, the low number of subjects makes it difficult to conclude about “prevalence” or generalize the findings. Also, the study is based on retrospective and self-reported data by nature. | Thank you for this comment. We agree that the data can be considered biased in some respects and have addressed the issue of self-selection bias in L. 464-475.  However, we have now added a paragraph considering retrospective self-reported accounts on L. 476-484, justifying this approach.  We are also not concerned with the prevalence of concussion in Rugby. We know that has a high prevalence, instead we were interested in how players think and feel towards concussion in amateur British Rugby. | L. 476-484 |
| The article title seems lengthy and unusual. | Thanks for this comment. We have amended the title now to ““I’ve not been knocked out, so I’ll probably be fine.” Amateur Rugby players do not know the risks they are taking”. | L.5-6 |
